# Early Detection and Prevention of Schizophrenic Psychosis—A Review

**DOI:** 10.3390/brainsci12010011

**Published:** 2021-12-23

**Authors:** Martin Lennart Schulze Westhoff, Johannes Ladwig, Johannes Heck, Rasmus Schülke, Adrian Groh, Maximilian Deest, Stefan Bleich, Helge Frieling, Kirsten Jahn

**Affiliations:** 1 Department of Psychiatry, Social Psychiatry and Psychotherapy, Hannover Medical School, D-30625 Hannover, Germany; praxis@dr-ladwig.com (J.L.); Schuelke.Rasmus@mh-hannover.de (R.S.); Groh.Adrian@mh-hannover.de (A.G.); Deest.Maximilian@mh-hannover.de (M.D.); Bleich.Stefan@mh-hannover.de (S.B.); Frieling.Helge@mh-hannover.de (H.F.); Jahn.Kirsten@mh-hannover.de (K.J.); 2 Institute for Clinical Pharmacology, Hannover Medical School, D-30625 Hannover, Germany; Heck.Johannes@mh-hannover.de

**Keywords:** early detection, epigenetics, psychosis, biomarkers

## Abstract

Psychotic disorders often run a chronic course and are associated with a considerable emotional and social impact for patients and their relatives. Therefore, early recognition, combined with the possibility of preventive intervention, is urgently warranted since the duration of untreated psychosis (DUP) significantly determines the further course of the disease. In addition to established diagnostic tools, neurobiological factors in the development of schizophrenic psychoses are increasingly being investigated. It is shown that numerous molecular alterations already exist before the clinical onset of the disease. As schizophrenic psychoses are not elicited by a single mutation in the deoxyribonucleic acid (DNA) sequence, epigenetics likely constitute the missing link between environmental influences and disease development and could potentially serve as a biomarker. The results from transcriptomic and proteomic studies point to a dysregulated immune system, likely evoked by epigenetic alterations. Despite the increasing knowledge of the neurobiological mechanisms involved in the development of psychotic disorders, further research efforts with large population-based study designs are needed to identify suitable biomarkers. In conclusion, a combination of blood examinations, functional imaging techniques, electroencephalography (EEG) investigations and polygenic risk scores should be considered as the basis for predicting how subjects will transition into manifest psychosis.

## 1. Introduction

In the quest to decipher the etiology and pathogenesis of schizophrenic psychoses, considerable progress was recently made with regard to the early detection of this spectrum of diseases [1,2].

Over the course of the last few years, numerous working groups primarily addressed the question of how people with an increased risk of developing schizophrenic psychosis could be identified at an early stage and what preventive measures could be adopted. This aspect is of great clinical relevance, as patients affected by a first psychotic episode (FPE) often report pre-existing psychological and social impairments, on average, for 5 years before the onset of the disease [3]. FPE manifests relatively late in terms of neurobiological development, usually around the age of 25 years [3]. At this time, however, abnormalities in brain structure, neurochemical processes, and brain connectivity have already become evident [4,5,6,7]. In addition, significant neuroanatomical, neurophysiological, neurocognitive, and neurohormonal changes were identified in people at risk of developing psychosis, which may likely support the transition process to manifest psychotic disorders [8,9,10,11]. Furthermore, a longer duration of untreated psychosis (DUP) seems to contribute to incomplete symptom remission, a higher likelihood of relapse, and a reduced quality of life [12]. Therefore, the timely detection of schizophrenic psychosis is of paramount importance. In addition to the established diagnostic methods of symptom description, e.g., clinical interviews and psychometric questionnaires (test batteries), it is hoped that neurobiological disease markers will simplify the prediction and assessment of schizophrenic psychosis. Epigenetic changes were widely described as crucial for the development and prediction of therapy response in many disease entities, e.g., in cancer or depression [13,14]. Their predictive value for the onset of psychotic symptoms was investigated by various groups, albeit with diverging results [15,16]. Synoptic conclusions have not been reached to date; therefore, the chief focus of this article is directed towards the discussion of the utility of various electrophysiological, imaging and molecular findings as potential biomarkers for the early detection of schizophrenic psychosis. Aside from epigenetics, other predictive strategies and possible treatment options will be considered.

## 2. Definition and Epidemiology of Schizophrenic Psychoses

The lifetime prevalence of schizophrenia was estimated at approximately 1% in various general population surveys and the point prevalence of schizophrenia was calculated as 0.28% in a large systematic review [17,18,19]. Schizophrenia is considered a severely debilitating disease with a high social burden and significant, far-reaching socioeconomic consequences for affected individuals [17,20]. The pathophysiological basis of schizophrenia, however, is still poorly understood. Clinically, schizophrenia is characterized by positive and negative symptoms. Positive symptoms include content-related thought disturbances, ego disturbances, and perceptual disturbances, whereas negative symptoms comprise anhedonia, social withdrawal, and cognitive and motor deficits such as blunted facial expressions and gestures [21]. The onset of schizophrenia peaks in adolescence or young adulthood, a time when the prefrontal cortex is still developing. The disease is typically preceded by a prodromal phase, which is defined by comparatively mild positive and negative symptoms that develop months to years before the onset of schizophrenia [22]. In the prodromal phase, affected persons display unspecific symptoms such as delusional mood, undirected fears or mistrustful experiences of relationships, all of which do not fulfill the definition criteria of schizophrenic psychosis [21,22].

## 3. Possibilities of Early Detection and Diagnosis by Operationalized Criteria and Structured Clinical Interviews

The path to the correct diagnosis of a schizophrenic psychosis is usually challenging due to several differential diagnoses that must be considered. In particular, autoimmune encephalitis or neurodevelopmental disorders evoked attention as psychotic symptoms are often initial manifestation signs of these diseases [23]. The consideration of only classical, first-rank symptoms of schizophrenia were associated with a false-positive and a false-negative rate of 5–20% and 40%, respectively [24]. For the prediction of initial psychotic manifestations, two significant groups of criteria were evaluated with respect to their prognostic quality: The ultra-high risk (UHR) and the basic symptom criteria. The UHR criteria, which were developed in the 1990s and continue to be widely used internationally, are based on studies by Yung and colleagues [25,26,27]. These criteria are part of several assessment scores or structural interviews to identify individuals who are at an increased risk of transitioning to psychosis. The UHR criteria include attenuated psychotic symptoms (APS) as well as short-lasting, so-called brief limited psychotic symptoms (remitting after a maximum of one week). Additionally, the UHR criteria also comprise genetic risk factors. If an individual meets the UHR criteria, this should be considered as an at-risk mental state (ARMS).

By contrast, the basic symptom concept was developed by Huber et al. in 1989 [27]. It describes self-perceived disturbances in several domains including disorders of drive and affect, thought and language processes, perception, proprioception, motor function, and central vegetative functions.

The main instruments used to assess an ARMS are the Comprehensive Assessment of At-Risk Mental States (CAARMS) and the Structured Interview for Prodromal Symptoms (SIPS) [28,29].

The criteria for the definite transition to a psychotic disorder are based, among others, on a definition by Yung et al. [30]: A transition is assumed if at least one positive symptom persists over a period of at least one week. Transition to a manifest psychotic disorder occurs in 8–54% of affected individuals within 1 to 2.5 years [26,31,32,33,34]. In a study conducted by Nelson and co-workers, individuals at UHR state were followed up for up to 14.9 years after initial presentation. In this study, the transition to a psychotic manifestation was observed in 34.9% of study participants. In all cases, transition to a manifest psychotic disorder took place within the first 10 years after initial presentation, and in two-thirds of cases occurred within the first 2 years after initial presentation [31]. Moreover, a large proportion of those who suffered a psychotic episode eventually developed full-blown schizophrenia [4].

## 4. Studying the Etiopathogenetic Hallmarks as a Basis for the Development of New Diagnostic Options in the Early Detection of Schizophrenic Psychosis

Despite several indicators of perturbed neurobiological development, to date, no direct trigger of schizophrenic psychosis is known [6,30,32,33]. The question of whether schizophrenia has an early developmental origin with prenatal onset or whether it should rather be considered a neurodegenerative disorder has not yet been fully elucidated. The two approaches are not mutually exclusive, however, and were reconciled by Keshavan and colleagues in their “two-hit hypothesis” [31,34]. The “two-hit hypothesis” postulates that a neural mismaturation during early development (“first hit”) predisposes to abnormalities in brain development during adolescence. Eventually, schizophrenic psychosis may arise under unfavorable (environmental) conditions (“second hit”).

## 5. Environmental Influences in Early Life

In addition to genetic factors that are subject to non-mendelian inheritance, pre- and perinatal environmental factors also contribute substantially to the development of schizophrenic psychosis. Interestingly, patients with schizophrenia were affected overproportionately by complications during birth. These include hypoxic events, complex cesarean section, preterm labor, and rhesus incompatibility [35,36]. As early as 1988, Mednick et al. described that children whose mothers suffered from influenza in the second trimester during the 1957 influenza epidemic were more likely to develop schizophrenia later in life [37].

**Figure 1:**
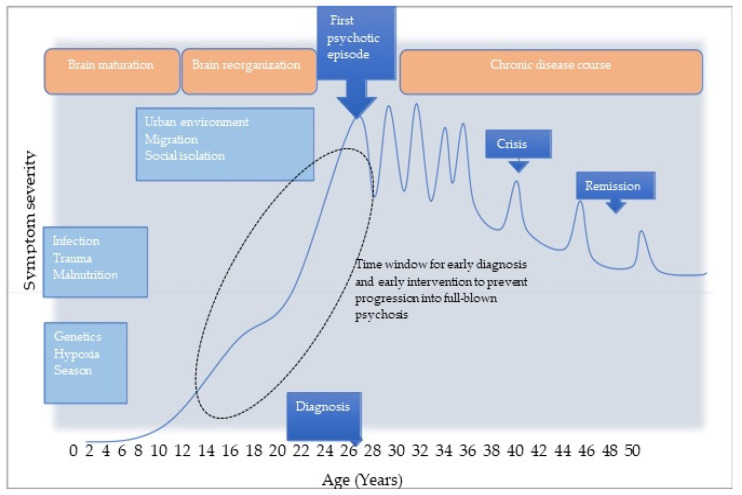
Development and course of psychotic disorders under the influence of environmental stimuli (adapted and modified from Millan et al. [44]).

Indirect influences, such as the season during which a child is born, also appear to have an impact. According to Mortensen et al., schizophrenia is 5 to 8% more common in those born in spring or winter [38]. In addition, there is a correlation between the size of a city in which an individual is born and the incidence of schizophrenia and other psychotic disorders. A clustering of schizophrenia cases was found in larger cities [39]. Other factors that may contribute to the development of schizophrenic psychosis include stress, substance abuse, social distress during childhood and adolescence, and social exclusion [40,41,42,43]. A schematic illustration of the development and the progression of psychotic disorders under the influence of various environmental risk factors is shown in Figure 1.

## 6. Genetics

The high heritability of schizophrenic psychosis was suggested by several twin studies. Genome-wide association study (GWAS) data further refined the understanding of the neurobiology underlying schizophrenic psychosis [45,46]. To date, a wide range of genetic risk variants (e.g., single-nucleotide polymorphisms (SNPs)) were described based on whole-genome analyses. Preceding this, numerous genomic abnormalities were studied in schizophrenia. Primarily, a balanced translocation disrupting two genes on chromosome 1 in a Scottish family was shown to be associated with the frequent occurrence of schizophrenia [47]. Since then, several candidate gene studies examined the involvement of genes associated with the metabolic processes of the central nervous system in the pathogenesis of schizophrenia. In various studies, only a few of them, such as *DISC1* (Disrupted-In-Schizophrenia 1), *COMT* (Catechol-O-Methyltransferase), *VMAT1* (Vesicular Monoamine Transporter 1) or *NRG1* (Neuregulin 1), could be sufficiently confirmed as relevant for schizophrenia development [48,49,50,51]. As their effect size seems to be relatively small, in the upcoming era of GWAS SNPs and copy number variants (CNVs) aroused great interest in the genetics of schizophrenia. One of the first findings was a correlation of deletions in *NRXN1* (Neurexin 1) gene and schizophrenia risk [52]. To date, around 12 different CNVs with genome-wide significance were reported to be functionally relevant with clear genome-wide significance [53]. Large-scale studies, especially those of the Psychiatric Genomics Consortium (PGC), characterized CNVs associated with a greater risk of schizophrenia as rare but very powerful, with a high relative genomic risk [54]. Additionally, genomic structural burden was higher in schizophrenia cases compared to controls [54]. On the contrary, SNPs contributing to schizophrenia risk are far more common, but with relatively small effect sizes [55]. As sample sizes have become considerably larger over recent years, more and more relevant SNPs could be identified. Groundbreaking in this context was a GWAS conducted by the Schizophrenia Working Group of the PGC, which found 128 SNPs linked to schizophrenia risk [56]. Nevertheless, recent research efforts led to the identification of more than 30 novel risk loci by involving even more participants not only of European, but also of Asian ancestry [57]. Since multiple genetic variants appear to contribute to the risk of developing schizophrenia, the use of polygenic risk calculations seems promising. In the context of the early detection of psychosis, the utility of polygenic risk scores was proven for predicting which UHR individuals will eventually develop psychotic symptoms [58]. Furthermore, risk scores provided evidence of a link between genetic liability to schizophrenia and brain structural changes or pharmacological treatment responses [59,60]. Thus, antipsychotic drugs demonstrated a higher efficacy in subjects with lower risk scores. Of particular interest, recent research efforts led to the development of a polygenic resilience score which describes the genetic basis of resistance to schizophrenia [61].

Since gene ontology analyses demonstrated an enrichment of risk variants in genes that regulate brain development, a neurodevelopmental origin of schizophrenia appears likely [62].

One study focused on transcriptional changes of UHR individuals during conversion to psychosis [63]. Intriguingly, the investigators detected an impairment in the expression of genes involved in the Wnt/ß-catenin pathway and Toll-like receptor signaling, suggesting an involvement of immune dysregulation in the onset of psychosis [63].

## 7. Epigenetics

There is evidence that noxious psychosocial and environmental stimuli influenced the risk of developing psychotic symptoms. The interaction of various factors in the development makes psychotic disorders multifactorial diseases. Furthermore, the clear influence of environmental processes suggests epigenetic mechanisms as a possible regulator [64]. With regard to schizophrenia, the promoter methylation of various genes was widely studied, more recently taking advantage of methylome-wide analyses [62,65,66]. Most of these studies included schizophrenic patients with an extensive disease course and multiple psychotic episodes in their clinical history, making it difficult to distinguish between the impact of previously administered antipsychotic medication on DNA methylation and pre-existing methylational changes [67]. To determine possible epigenetic risk constellations as indicators for the transition from a UHR state into a manifest psychotic disorder, studies including UHR individuals, people with familial risk states, and FPE patients are of particular interest. Unfortunately, research in this field is scarce with only limited data available. To the best of our knowledge, thus far, only four studies have investigated epigenetic processes in people with preclinical symptoms or with childhood psychotic experiences [15,16,68,69]. All of these studies exhibit a longitudinal study design, which has several advantages compared to the cross-sectional study design predominantly applied in schizophrenia research. Roberts and co-workers reported that the hypomethylation of a CpG site linked to genes *C7orf40* and *SNORA9* was associated with the persistence of psychotic experiences from childhood to adolescence [15]. As a limitation of this study, it must be mentioned that the inclusion criteria did not clearly differentiate between individuals with stand-alone psychotic experiences and participants with a definitive UHR state. This aspect, by contrast, was addressed in another study that compared the methylome of UHR individuals who developed a full-blown psychosis to those who did not within a follow-up period of one year [16]. Two differentially methylated regions (DMRs) could be identified, one of them located in the promoter of the *GSTM5* gene. The encoded protein, glutathione S-transferase mu 5, plays an important role in protective mechanisms against oxidative stress. A cluster analysis of CpG sites with the most significant methylation changes during psychotic conversion additionally suggested an involvement of oxidative stress mechanisms. Furthermore, an involvement of axonal guidance processes and inflammation was suggested. The same study population as in ref. [16] served as basis for the analysis of intraindividual methylomic variability in another study [68]. Methylomic variability was suggested as a contributing factor in the development of various somatic and psychiatric diseases. Kebir et al. proposed the methylomic instability of two genes whose encoded proteins are implicated in sphingolipid pathways in conversion to psychosis [68]. Another approach to identify epigenetic biomarkers was the comparison of the methylome of monozygotic twins discordant for psychotic symptoms during childhood [69,70]. The most significant DMR was located in the promoter of the *C5orf42* gene [69]. The gene function has not yet been clearly deciphered, but mutations were linked to Joubert syndrome, a neurodevelopmental disorder [71]. By analyzing evidence of methylomic studies involving UHR individuals together, there does not appear a clear trend of a causal pathway for disease initiation. A complex interplay of dysregulations in inflammatory factors and cellular energetic processes seems likely.

Not only whole-methylome analyses but also candidate gene studies are sparse in the context of pre-clinical psychosis. The most interesting finding was that the methylation of the oxytocin receptor gene was significantly decreased before and after the development of a full-blown psychosis and could be linked to typical negative symptoms [72]. Moreover, for the understanding of the onset of psychotic symptoms, the study of FPE patients could be useful. Regrettably, only a single whole-methylome study focused on this patient population [73]. Nishioka and colleagues demonstrated a global hypomethylation in FPE patients [73]. Differentially methylated CpG sites were markedly enriched in genes associated with the constitution of intracellular organelles or transcription factor binding, but DMRs could also be detected in promoters of the *HTR1E* and *COMTD1* genes, which were both clearly linked to the development of schizophrenia [73]. There are few studies available that examine the methylation of candidate genes in FPE patients. The most interesting findings among these were a hypomethylation within *GRIN2B* promoter and an increased methylation of *GCH1* promoter in FPE patients [74,75]. While the protein encoded by *GRIN2B* is involved in glutamate signaling, *GCH1* is crucial for the synthesis of several neurotransmitters [74,75].

As environmental factors are crucial for development of psychosis, the influence of various risk factors on epigenetics, in individuals with FPE or at the UHR state, is of special interest. Adverse childhood experiences were proven to decrease the methylation of *FKBP5* whose gene product, FK506 binding protein 5, is crucial for glucocorticoid receptor signaling [76]. Furthermore, hypomethylation in general was associated with childhood trauma in psychotic patients [77]. More data regarding the influence of schizophrenia-related risk factors on DNA methylation are available, but these studies did not focus on psychiatric patients.

In conclusion, no clear trend could be identified to determine which systems were most important with regard to epigenetic mechanisms in UHR individuals transitioning to full-blown psychosis. Indeed, the participation of widely described factors in psychotic diseases, such as inflammatory response, energetic pathways, neuromodulation, or neurotransmitter synthesis, appears likely. While epigenetic mechanisms, especially DNA methylation, are certainly implicated in the transition to psychosis, there is only a small overlap between the studies conducted so far with regard to possibly involved genes. In this respect, larger population-based studies that include individuals with pre-clinical symptoms are urgently warranted.

## 8. Magnetic Resonance Imaging

Evidence suggests that cortical networks are reorganized during adolescence. It is known that there is a physiological loss of synaptic density of about 30% during adolescence in the dorsolateral prefrontal cortex. Patients suffering from schizophrenic psychosis display a higher reduction of about 60% [78,79]. Therefore, it is believed that even after the fetal and perinatal period, the disturbances in neuronal development may occur and promote the development of schizophrenic psychosis, and that this disruption of neuronal development results in the fronto-temporal gray matter volume (GMV) reduction seen in UHR individuals [8,80]. Indeed, numerous early computed tomography (CT) as well as magnetic resonance imaging (MRI) studies demonstrated morphological brain alterations in people with schizophrenia [81,82,83,84]. The most common findings were the dilatation of the ventricular system and GMV reduction, especially in the cortical and subcortical gray matter of the frontal lobe, temporal lobe, and limbic system [85,86,87,88,89]. Interestingly, these changes do not appear to be static but are subject to a progressive process [90,91]. Meanwhile, studies revealed functional and anatomical changes in cortical areas in individuals even before the onset of psychosis [92,93]. Differences in individual brain regions that were dependent on the further course of the disease could be observed [94,95]. While reductions in GMV of the parietal, medial temporal, and inferior frontal cortex were observed in UHR individuals who later developed schizophrenia, a reduced volume of the subcallosal cingulate was primarily detected in individuals who later developed affective psychosis. Reduced volumes of the amygdala and insula, by contrast, were primarily registered in UHR individuals who later developed bipolar disorder [94,95]. When comparing high-risk subjects who transitioned to psychosis with high-risk individuals who did not, the former group was characterized by reduced GMV of the prefrontal cortex (and specifically the orbitofrontal cortex), the temporal cortex (in particular, the medial temporal gyrus), and the cerebellum [96,97,98]. Furthermore, GMV reduction was also reported for limbic system structures—(anterior) cingulate cortex, insula, and hippocampus—albeit less frequently [96,97]. Concerning the predictive value of white matter (WM) integrity, thus far, evidence has been inconclusive. Some studies found reduced WM (e.g., in the left superior temporal lobe or globally) as well as increased WM (e.g., in the frontal lobe and the left medial temporal lobe) in UHR subjects who transitioned to psychosis [99,100,101]. Other studies, however, did not observe a significant difference in WM integrity between UHR subjects who developed psychosis and those who did not [102].

Whereas structural MRI was frequently applied to investigate the potential predictors of the transition to psychosis, there is less evidence from functional MRI (fMRI) studies. An early study found that subjects at a genetically high risk, who later transitioned to schizophrenia, displayed increased parietal and decreased anterior cingulate activity, as well as smaller activation increases with higher cognitive demands in bilateral, temporal regions and right lingual gyrus, during a sentence completion task [103]. Importantly, predictive tests based on the parietal lobe and the lingual gyrus were able to discriminate between those who transitioned to schizophrenia and those who did not, with positive and negative predictive values of 0.8 and 1.0, respectively. Since fMRI is an elegant tool to explore brain connectivity, it is well-suited to investigate the dysconnectivity hypothesis of schizophrenia [6,104]. Van den Heuvel et al. showed that the physiological “rich club organization” of brain networks—the phenomenon that the most highly interconnected brain regions are also highly interconnected among themselves—is markedly disturbed in schizophrenia [104,105]. Various following studies that compared young adults with schizophrenia and healthy controls by means of structural diffusion tensor imaging and resting-state fMRI replicated the perturbation of the “rich club organization” as an early event in schizophrenia development [106,107,108]. Of note, a structural disorganization of brain connectivity was already shown in individuals who are at an increased risk of developing psychosis. Therefore, it is discussed whether abnormal “rich club organization” could represent an endophenotypic marker for psychosis onset [109]. Indeed, UHR individuals who transitioned to psychosis did not only display an increased activation of bilateral prefrontal cortex, brainstem, and left hippocampus during a phonological fluency task, but also increased connectivity between the prefrontal cortex and midbrain [110]. Finally, hypoconnectivity between the thalamus, prefrontal cortex, and cerebellum, as well as hyperconnectivity of the thalamus and sensory motor areas during resting state fMRI, were also found to be differentiating features between UHR subjects who transitioned to psychosis and those who did not [111].

Traditional statistical analytic approaches may identify group differences, but they do not allow for reliable predictions on a single-subject level (i.e., predict whether a UHR individual will transition to psychosis or not). Therefore, in recent years, many studies have probed machine-learning techniques in an effort to derive predictive models based on neuroimaging data, which may aid clinicians in diagnosing psychiatric diseases. With regard to schizophrenia, supervised learning models in the form of support vector machines are the most widely employed technique, achieving within-study accuracies of about 75–90% for differentiating between patients and healthy controls [112]. Support vector machines demonstrated similar accuracies (80–88%) for predicting the transition to psychosis in UHR individuals, with high positive (78–100%) and negative (80–90%) predictive values [113,114,115,116]. However, given the relatively small sample sizes, overfitting in these studies is likely, and such algorithms still need to be validated in new samples prior to widespread clinical applications. In the future, the combination of genetic, epigenetic, and neuroimaging data may prove particularly effective for improving predictions at the subject level, as has already been demonstrated for SNP and fMRI data [117].

Despite promising findings, it must be cautioned that MRI measures such as GMV, which rely on voxel-based morphometry or diffusion tensor imaging, are susceptible to motion artifacts and may have previously been systematically confounded by factors such as medication, smoking status, medical and psychiatric comorbidities, and metabolic state. Of note, the very regions where volume reductions were detected in UHR individuals who transitioned to psychosis are particularly liable to confounding [118].

## 9. Marker-Specific Imaging as Indicator for Disturbed Neurotransmission

Currently, there are various tools available to study disturbances in neurotransmitter systems regarding the transition to psychosis. These techniques comprise positron emission tomography (PET), single-photon emission computed tomography (SPECT), and magnetic resonance spectroscopy (MRS). Research especially focused on alterations of the dopamine system. This was due to the oldest and most established theory on the pathogenesis of schizophrenia: The “dopamine hypothesis”. This hypothesis postulates that an overactivity of certain dopaminergic brain regions is especially responsible for the positive symptoms of schizophrenia. This notion is clinically supported by the observed symptom improvement in patients treated with dopamine receptor antagonists (i.e., antipsychotics). Elevated dopamine synthesis capacity in the striatum or brainstem was linked to a higher risk of transition to psychosis in UHR individuals in several studies [110,119,120]. Furthermore, disturbances in the dopamine synthesis of UHR subjects was linked to changes in the performance of special cognitive tasks [110,121]. The second, recent transmitter system of special interest is the glutamatergic system. MRS studies revealed that higher glutamate levels in precommissural dorsal-caudate preceded the onset of psychosis [119,122]. The development of full-blown psychosis was also accompanied by reduced glutamate or glutamine concentrations in thalamic regions and elevated concentrations in the prefrontal cortex and striatum [122,123,124,125]. A link between region-specific glutamatergic and dopaminergic activity in the hippocampus and striatum in UHR subjects was hypothesized; however, a combined PET/MRS study by Howes et al. failed to prove a correlation [126].

## 10. Further Possible Biomarkers

It is hoped that suitable biomarkers may be identified that aid in the diagnosis, prognosis, and creation of individualized treatment plans for patients suffering from schizophrenia. The approach of some works is based on the hypothesis that mental illnesses are systemic disorders and that changes might, therefore, be found in systems other than the central nervous system, for example in the circulation. The process of identification of proteomic biomarkers has revealed changes in inflammatory, hormonal, and metabolic pathways in patients with schizophrenia [127]. In particular, an inflammatory cascade is believed to play a role in the initiation process of FPEs. Nevertheless, data on whether pro- or anti-inflammatory processes predominate in people with ARMS are conflicting. In general, elevated levels of interleukin (IL-)1ß, IL-7, and IL-8 were reported to indicate the transition from an ARMS to a manifest psychotic episode [128]. Furthermore, elevated levels of tryptophan metabolism appear to correlate with increased inflammation in schizophrenia, suggesting that immune dysregulation may also involve the kynurenine pathway [129].

## 11. Electroencephalography

Electroencephalography (EEG) studies have shown promising results. The “mismatch negativity” (MMN), a component of event-related potentials (ERPs) evoked by an odd stimulus in a sequence of stimuli, shows a consistent and robust reduction in patients with schizophrenia and could be useful as a biomarker since the changes are present even before the onset of psychosis [130,131,132]. MMN changes are widely registered as correlates of disturbed informational processing under several neuropathological conditions, especially in Alzheimer’s disease [133]. To date, reduced MMN is the most reliable EEG biomarker distinguishing UHR individuals who transition to schizophrenia from those who do not [131,132,134]. Comparable to MMN, P300 is another ERP component that is also evoked by unexpected stimuli in oddball paradigms; however, P300 is only evoked when stimuli are actively attended to. P300 abnormalities are a common finding in schizophrenia and were also found in unaffected relatives, leading to the proposition that P300 may in fact be an endophenotype of the disorder [135,136,137]. Indeed, amplitude decreases in P300 were found to predict the transition to psychosis in UHR individuals, not only when evoked by auditory but also by visual oddball stimuli [9,138]. In the future, EEG microstates—transient, quasi-stable patterns of EEG activity typically lasting tens to hundreds of milliseconds—may also be useful for predicting the transition to psychosis in UHR subjects. In a pioneering study, de Bock et al. demonstrated that microstate D was reduced in UHR subjects who later developed psychosis in comparison to those who did not, as assessed by the coverage of its temporal characteristics, duration, and occurrence [139,140]. The importance of the temporal features of microstate D for differentiating between first-episode patients, UHR individuals, high-risk individuals, and healthy controls was confirmed by another study, achieving a within-group classification accuracy, sensitivity, and specificity of 92%, 91.8%, and 90.8%, respectively, by using a random forest model and combining microstate, behavioral, and demographic features [141]. This study impressively illustrates that machine-learning approaches, which combined data from different modalities—including EEG data—bear great promise for improving predictions of the transition to psychosis in the coming years.

## 12. Early Therapeutic Options

The ultimate goal of early detection strategies is to prevent the transition into full-blown psychosis. Therefore, the effectiveness of cognitive behavioral therapy and antipsychotic medication is intensively studied [142,143,144,145,146].

Whether pharmacotherapy alone can delay or even prevent a transition to a psychotic disorder has not yet been established. McGorry et al. were the first to study the use of the second-generation antipsychotic risperidone and additional cognitive-oriented psychotherapy in UHR individuals [143]. They found an early advantage for the intervention group with respect to conversion to psychosis, which was, however, no longer present 6 months after treatment discontinuation [143]. In the following period, several other groups found mild positive effects of cognitive-oriented psychotherapy, family-based therapy, or antipsychotics, while others did not [144,145,146,147,148].

Even though favorable effects on positive and negative symptoms, as well as on cognitive functions were demonstrated, antipsychotic medication is currently not routinely recommended for the ARMS period [142,149,150,151,152,153,154]. Antipsychotics should only be considered for a limited period of time if deemed clinically necessary, e.g., to achieve sufficient symptom relief for psychotherapeutic interventions [155].

Since decreased omega-3 fatty acid levels are present in patients with schizophrenia as well as in UHR individuals, it is hoped that the administration of omega-3 fatty acids will have a preventive effect and improve symptoms, despite currently inconclusive efficacy data [156,157,158]. A recent study did not show a benefit of omega-3 fatty acids for UHR individuals [159].

As the intervention strategies described above appear to have, at best, small effect sizes, new approaches are being discussed. These include, for example, the use of cannabidiol or the administration of amino acids such as glycine or serine, and displayed promising results in smaller clinical studies [160,161,162]. However, further studies are needed to corroborate these findings.

## 13. Conclusions

Over the course of the last 20 years, our knowledge about the neurobiology of the UHR state and the prodrome of a first psychotic disorder has significantly increased. It is widely accepted that the duration of untreated psychosis (DUP) is of paramount importance for the subsequent disease course. Prolonged DUP is associated with incomplete symptom remission, a higher likelihood of relapse, and a reduced quality of life [12]. To minimize the DUP, screening approaches during childhood were considered and several school-based screening campaigns were validated [163,164]. Moreover, we also call for detection strategies at universities. Many research groups addressed the question of how to identify individuals at increased risk of developing a psychotic disorder at an early stage.

According to the current literature on diagnostic methods for the detection of a high-risk stage, only about 30% of affected individuals with pre-clinical symptoms develop a psychotic disorder in the further course [8,165,166].

Even though a large proportion of individuals in a high-risk state will eventually not develop a psychotic disorder, longitudinal studies showed that high-risk patients already continuously exhibit mild psychotic symptoms and lower levels of functioning compared with healthy controls [167,168]. High-risk individuals also more likely demonstrate persistent psychopathology and a need for treatment, regardless of the transition rate [169,170]. Symptoms of depression and anxiety disorders are common, as are disturbances in the sleep–wake cycle [171]. The presence of a UHR state can also be a substantial burden for the affected person and is sometimes accompanied by conflicts in family and social environments. Among individuals who do not develop a psychotic disorder, a wide variety of courses are described. Whereas some individuals show a complete remission of symptoms, others develop additional disorders, mostly depression, anxiety disorder, addiction, or bipolar disorder [167].

In this article, we compiled evidence about possible biomarkers that hold promise for differentiating between UHR individuals who will transition to full-blown psychosis from those who will not. As environmental influences seem to be crucial for the development of manifest psychotic disorders, epigenetic processes potentially act as a mediator between individual risk factors and genomic patterns. Indeed, to identify reliable epigenetic markers, larger study cohorts are needed. Moreover, not only large cohort studies are required for exploring new biomarkers, but also different methodological approaches. Last but not least, large clinical datasets should be analyzed with the help of bioinformatics to identify suitable biomarkers.

Transcriptomic, methylomic, and proteomic studies indicate an involvement of immune dysfunction in the processes underlying the development of full-blown psychosis [16,63,128]. This appears a promising research target, which may open the door for new therapeutic options such as immunomodulatory agents. Due to the non-mendelian inheritance, it appears unlikely to identify a single diagnostic method with sufficient sensitivity and specificity. Therefore, we recommend a combination strategy of blood examination, polygenic risk scores, functional imaging approaches, and EEG investigations.

Early supportive therapies, e.g., cognitive psychotherapy or the administration of omega-3 fatty acids are being intensely discussed, but it is not yet clear what the best therapeutic regimen might be [155]. Antipsychotic medication is currently not routinely recommended for the ARMS period [142].

In conclusion, the reliable early detection of psychotic illness remains challenging to date. Although psychometric tests allowing predictions are available, their application in clinical routine is rather complex. Furthermore, there are only a few specialized centers dedicated to the early detection of schizophrenic psychosis. Great hope is invested in biomarkers that will facilitate diagnostics, prognostics, and the creation of individualized treatment plans [172,173]. For a better understanding of the initiation process of psychosis, we strongly advocate for the conduction of larger population-based studies.
